# Cytoplasmic free Ca^2+^ is essential for multiple steps in malaria parasite egress from infected erythrocytes

**DOI:** 10.1186/1475-2875-12-41

**Published:** 2013-01-30

**Authors:** Svetlana Glushakova, Vladimir Lizunov, Paul S Blank, Kamran Melikov, Glen Humphrey, Joshua Zimmerberg

**Affiliations:** 1 Program in Physical Biology, Eunice Kennedy Shriver National Institute of Child Health and Human Development, Bethesda, MD, 20892, USA

**Keywords:** *Plasmodium falciparum*, Asexual cycle of replication, Parasite egress, Free calcium, Swelling of parasitophorous vacuole

## Abstract

**Background:**

Egress of *Plasmodium falciparum,* from erythrocytes at the end of its asexual cycle and subsequent parasite invasion into new host cells, is responsible for parasite dissemination in the human body. The egress pathway is emerging as a coordinated multistep programme that extends in time for tens of minutes, ending with rapid parasite extrusion from erythrocytes. While the Ca^2+^ regulation of the invasion of *P. falciparum* in erythrocytes is well established, the role of Ca^2+^ in parasite egress is poorly understood. This study analysed the involvement of cytoplasmic free Ca^2+^ in infected erythrocytes during the multistep egress programme of malaria parasites.

**Methods:**

Live-cell fluorescence microscopy was used to image parasite egress from infected erythrocytes, assessing the effect of drugs modulating Ca^2+^ homeostasis on the egress programme.

**Results:**

A steady increase in cytoplasmic free Ca^2+^ is found to precede parasite egress. This increase is independent of extracellular Ca^2+^ for at least the last two hours of the cycle, but is dependent upon Ca^2+^ release from internal stores. Intracellular BAPTA chelation of Ca^2+^ within the last 45 minutes of the cycle inhibits egress prior to parasitophorous vacuole swelling and erythrocyte membrane poration, two characteristic morphological transformations preceding parasite egress. Inhibitors of the parasite endoplasmic reticulum (ER) Ca^2+^-ATPase accelerate parasite egress, indicating that Ca^2+^ stores within the ER are sufficient in supporting egress. Markedly accelerated egress of apparently viable parasites was achieved in mature schizonts using Ca^2+^ ionophore A23187. Ionophore treatment overcomes the BAPTA-induced block of parasite egress, confirming that free Ca^2+^ is essential in egress initiation. Ionophore treatment of immature schizonts had an adverse effect inducing parasitophorous vacuole swelling and killing the parasites within the host cell.

**Conclusions:**

The parasite egress programme requires intracellular free Ca^2+^ for egress initiation, vacuole swelling, and host cell cytoskeleton digestion. The evidence that parasitophorous vacuole swelling, a stage of unaffected egress, is dependent upon a rise in intracellular Ca^2+^ suggests a mechanism for ionophore-inducible egress and a new target for Ca^2+^ in the programme liberating parasites from the host cell. A regulatory pathway for egress that depends upon increases in intracellular free Ca^2+^ is proposed.

## Background

Malaria parasite replicates within its own plasma membrane, positioned inside a parasitophorous vacuole that is located within the host human erythrocyte. Both the vacuolar and erythrocyte plasma membranes must be breached by the parasites to exit the host cell (see reviews by V Lew [1,2]. Experimental evidence supports a multistep process [3-9] involving at least two Ca^2+^-dependent enzymes: protein kinase PfCDPK5 [5] and the host protease calpain [4]. The emerging complexity of the egress mechanism suggests a high degree of spatial and temporal coordination. Cytosolic Ca^2+^ is a universal secondary messenger for intracellular signalling in eukaryotic cells (for review see [10]). Ca^2+^ signalling in malaria parasites is well recognized but poorly understood; several signalling pathways have been described [11-14]; for a recent review see [15]. The total erythrocyte calcium content increases with infection [16,17]. However, it is harder to determine the free calcium concentration, [Ca^2+^, in the various compartments of an infected erythrocyte at the schizont stage of the asexual cycle. Divided and tightly packed parasites occupying approximately half of the erythrocyte volume [18], are situated within a series of concentric membrane spheroids; the individual parasites have additional levels of membrane compartments (organelles) internal to their own plasma membrane. Literature estimates of [Ca^2+^ in different compartments range from low nM in the erythrocyte cytoplasm [19] to ~ 40 μM in the parasitophorous vacuole – the space between the PV membrane and the parasite plasma membrane [13]. Presumably, there are compartments within the parasite that maintain even higher Ca^2+^ concentrations and can act as intracellular Ca^2+^ stores [11]. Thus, there is a complex set of potential gradients for Ca^2+^ translocations between compartments with differing [Ca^2+^. An influx of extracellular Ca^2+^ into erythrocytes is required for parasite invasion [20-22], however, the role Ca^2+^ plays in the multistep egress programme is not clear [21,23]. The role of intracellular Ca^2+^ is intensely studied in the egress programme of the related *Apicomplexan* parasites, *Toxoplasma*[24-29]. However, sufficient differences in the molecular mechanisms of the egress pathway between this species and the malaria parasites are emerging [28,30]. This study reports the measurement of intracellular fluorescent probes for [Ca^2+^ during the egress pathway of *Plasmodium falciparum* from infected erythrocytes. Perturbation with calcium homeostasis reagents together with quantitative measures of egress that decouple egress from invasion allowed a determination of one source for the calcium fluxes that must occur for [Ca^2+^ to change. Finally, to determine which of the observable stages of the parasite egress programme [7] are sensitive to changes in [Ca^2+^, the morphology of treated cells was analysed.

## Methods

### Culture of *Plasmodium falciparum*

*Plasmodium falciparum* strain 3D7 (ATCC, Manassas, VA, USA) was cultured, according to the Trager–Jensen method [31], with a modification that uses a controlled gas mixture (5% CO_2_, 5% O_2_, 90% N_2_) instead of a candle jar, in human erythrocytes in RPMI 1640 medium (Invitrogen) supplemented with 25 mM Hepes (Invitrogen), 4.5 mg ml^−1^ glucose (Sigma), 0.1 mM hypoxanthine (Invitrogen), 25 μg ml^−1^ gentamicin (Invitrogen) and 0.5% AlbuMax II (Invitrogen). Schizonts were isolated from infected cultures using Percoll enrichment and used to initiate a new 4 h-span, synchronized infection [32,33] in erythrocytes from donor blood drawn within three days of the procedure.

### Parasite egress assay

The detailed description of the parasite egress assay is published [34]. Briefly, experimental and control parasite-infected cells, approaching the end of the cycle, were injected together with their experimental or control solutions into separate microscopy chambers and incubated at 37°C for an interval of time specified for each experiment, before cooling chambers to 15°C to end egress. Egress was calculated as the fraction of schizonts ruptured in the chamber during the incubation time. Treatment effect on parasite egress was evaluated by comparing the fraction of ruptured schizonts in treated and control cultures. Calcium ionophore A23187, EDTA, EGTA, staurosporine, cyclopiazonic acid, and thapsigargin was purchased from Sigma-Aldrich (St. Louis, MO, USA); BAPTA AM, calcein AM, Fluo-4 AM, Fura Red AM and ethidium homodimer were purchased from Invitrogen (Eugene, OR, USA). Cell suspensions of infected erythrocytes at 0.5% haematocrit in AlbuMax II-containing medium were used.

### Live cell microscopy

Laser scanning microscopy (LSM 510, Zeiss; 100x or 63x 1.4 NA oil objectives) was used to follow the morphology of parasite-infected erythrocytes, to detect labelled viable or dead parasites, and the origin of swelled membranes in ionophore-treated cultures. To discriminate between the erythrocyte and vacuolar membranes, abundant proteins associated with the erythrocyte membrane were detected using fluorescence microscopy. Glycophorin A was detected using a fluorescent antibody (Allophycocyanin anti-CD235a, BD Biosciences, San Jose, CA, USA) and the cytoskeletal protein F-actin was detected using fluorescent phalloidin (Alexa Fluor 488 phalloidin, Invitrogen, Eugene, OR, USA) as described in [7]. Allophycocyanin and Alexa Fluor 488 were excited at 514 nm and 488 nm respectively. Calcein was excited at 488 nm; ethidium homodimer was excited at 514 nm. Differential interference contrast microscopy was performed using 488 nm laser illumination. To detect free calcium, infected red blood cells were labelled with the calcium indicator Fluo-4 AM (5 μM) and were imaged in time using 488 nm laser illumination; acquisition times varied from ~0.7–31 seconds per image frame. Using ImageJ (NIH) the image sequences were background subtracted (Rolling Ball, 75 pixels), a region of interest that included only the infected cell defined, and the mean fluorescence intensity per pixel calculated as a function of time. The resulting time series were normalized by the initial value: (F(t)-F(t = 0))/F(t = 0) or ΔF/F.

Long-term experiments with Fura Red AM-labelled cells to detect changes in free calcium level were performed in an environmental chamber at 37°C using an inverted fluorescence microscope (Nikon Ti) equipped with 60× 1.49 NA objective, TIRF-illumination arm, and custom-built laser combiner (405, 488, 561 and 640, Coherent). Cells were labelled 30 min with 5 μM Fura Red AM at 37°C in the medium supplemented with 40 μM of probenecid, washed and resuspended to 0.5% haematocrit in medium with 40 μM probenecid. Sequential illumination using 405 nm and 488 nm lasers was used to excite Fura Red. The incident angle of the laser beam was set by a motorized TIRF-unit to wide-field illumination (90 degrees). Fluorescence signals were separated from the excitation light using a quad-band dichroic and emission filter set (405/488/561/640, Semrock) and digitized using an Andor Ixon EMCCD camera. Twenty-four to 48 frames were acquired for each fluorescence channel using a 200 ms exposure and a 5-min interval between exposures to avoid cell photodamage. Microscope, laser, and camera were controlled using Micro-Manager 1.4.10 [35]. Fluorescence images were processed using a custom ratio-metric macro (available upon request) in ImageJ (NIH). In brief, the image sequences were background subtracted (Rolling Ball, 75 pixels), and aligned to correct for cell motion and drift. A region of interest that included only the infected cell was selected, and the mean fluorescence intensity per pixel was calculated as a function of time. The ratio of fluorescence F_405_/F_488_ was calculated for each infected cell from background-subtracted images. The F_405_/F_488_ ratio rate of change (dRatio_405_/_488_/dt) was estimated as the difference between final and initial ratio values divided by the duration of the growth period. Calcium estimates of the ratio measurements were derived using Monte Carlo simulations. The relationship [Ca^2+^ = Kd*F_488_([Ca^2+^_min_)/F_488_([Ca^2+^_max_)*(R-R_min_)/(R_max_-R) was used to estimate the ratio R using average values and standard deviations for F_488_([Ca^2+^_min_), F_488_([Ca^2+^_max_), R_min_ and R_max_ where the lowest and highest ratio values and fluorescence intensities from early and late stage schizonts were used as approximations in lieu of direct calibration. Each ratio value at a specific calcium concentration was the average of 100,000 simulations of normal random variables calculated using the population averages and standard deviations, repeated 100 times. A Kd of 0.7 μM was used for Fura Red and represents the expected increase in Kd in the cellular environment [36,37] compared to the saline value of 0.14 μM.

## Results and discussion

### Changes in intracellular [Ca^2+^] in late-stage infected cells

**Figure 1:**
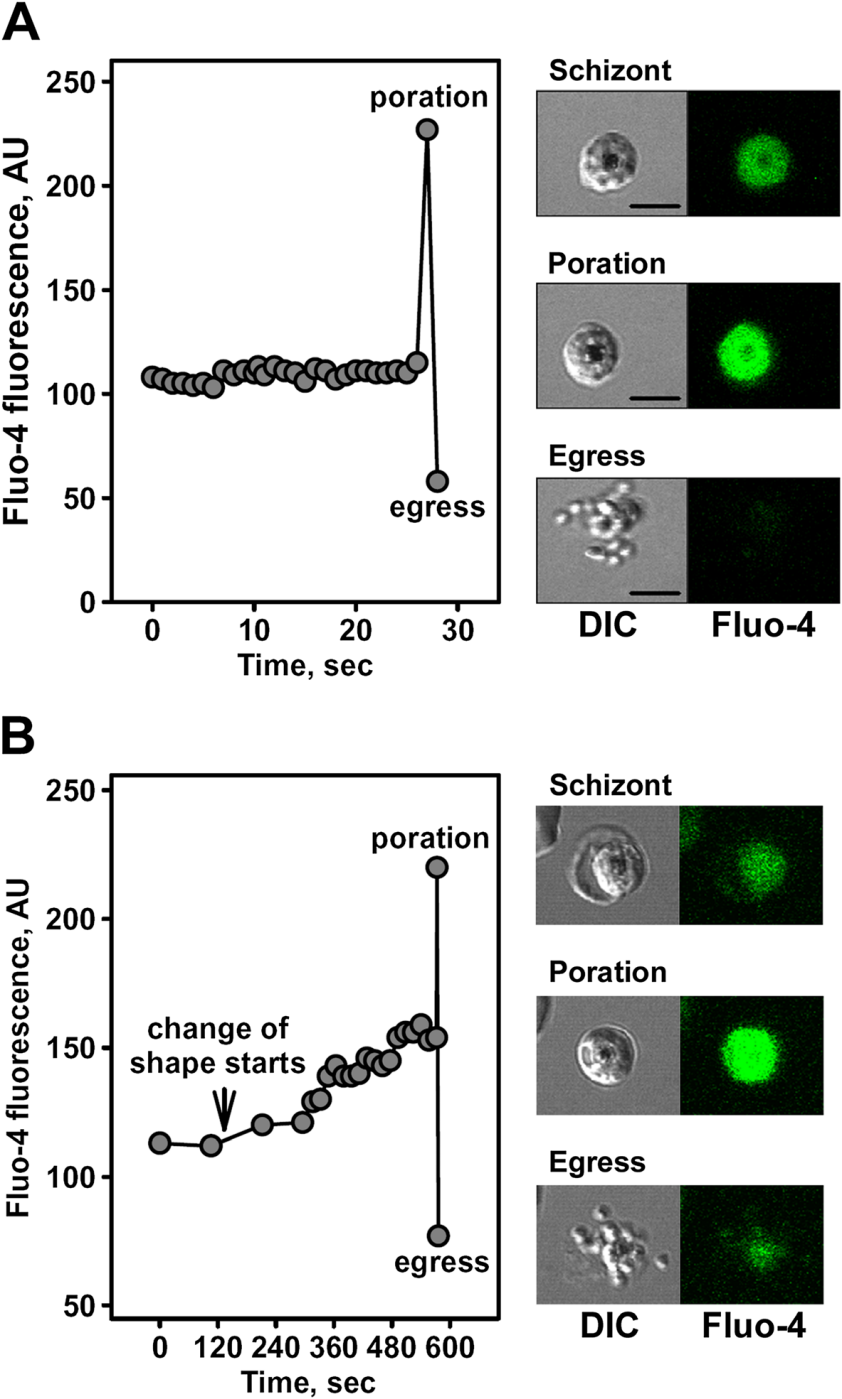
**Free calcium kinetics in schizonts approaching parasite egress.** Cells labelled with Fluo-4 AM (5 μM), and monitored at 37°C in full medium **(A)** or full medium supplemented with 40 μM probenecid **(B)**. Selected frames from the time-lapse movies on the right side of each graph show DIC and fluorescence images of pre-egress schizonts (upper set of images), pre-egress schizonts with the erythrocyte membrane permeable to external calcium (middle set of images labelled as ‘poration’ showing the highest level of fluorescence signal) and lower set of images captured erythrocyte membrane rupture and parasite egress. Bar = 5 μm.

To detect intracellular Ca^2+^ signalling in the parasite and erythrocyte prior to egress, mature schizonts were labelled with the Ca^2+^ probe Fluo-4 AM and then imaged using short-term and low-intensity 488 nm illumination to avoid photodamage [6]. Because short transients (spikes) in [Ca^2+^ are seen in other systems in which Ca^2+^ triggers physiological pathways (reviewed in [38]), an imaging strategy aimed at detecting transients was used. A large increase in Fluo-4 fluorescence, spatially averaged over the infected erythrocyte, was observed in 10 of 14 image sequences prior to parasite egress (Figure 1A, Additional file 1). The magnitude of the fluorescence increase in ΔF/F varied from 0.22–1.84 (0.66 ± 0.16, Mean ± SEM) and relaxed with egress itself. The time between this transient peak and parasite egress varied from 0.98–10.63 sec (3.48 ± 1.28 sec, mean ± SEM). This change in fluorescence is consistent with an influx of extracellular Ca^2+^ (internal [Ca^2+^ likely peaking at the level that saturates Fluo-4 affinity for Ca^2+^) into the erythrocyte through parasite-induced pores that are produced a few seconds prior to parasite egress within the host membrane, presumably by perforins [7] previously detected in schizonts [39]. To confirm this conclusion, extracellular Ca^2+^ was depleted using a Ca^2+^ chelator and parasite egress was recorded in Ca^2+^-free medium. No intracellular spike in Fluo-4 fluorescence prior to egress was observed in three of three recordings of parasite egress. Thus, the ability to measure changes in intracellular [Ca^2+^ in the erythrocyte cytoplasm of late schizonts was confirmed. Next, the records of Fluo-4 fluorescence occurring prior to this poration signal were analysed but no [Ca^2+^ spikes were detected. Possible reasons for failing to detect calcium spikes are: a) their duration is less than the instrument temporal resolution; b) the calcium transient amplitude is below the instrument detection limit. [Ca^2+^ transients of the duration, amplitude, and compartmental location comparable to the poration-induced spike do not occur during the egress pathway.

To test the hypothesis that a quasi-static Ca^2+^ concentration is necessary to activate Ca^2+^-sensitive proteins regulating or affecting the egress pathway, another protocol to probe [Ca^2+^] at longer time scales was adopted. To avoid cell photodamage, the time of observation was extended but the interval between frames was increased.

**Figure 2:**
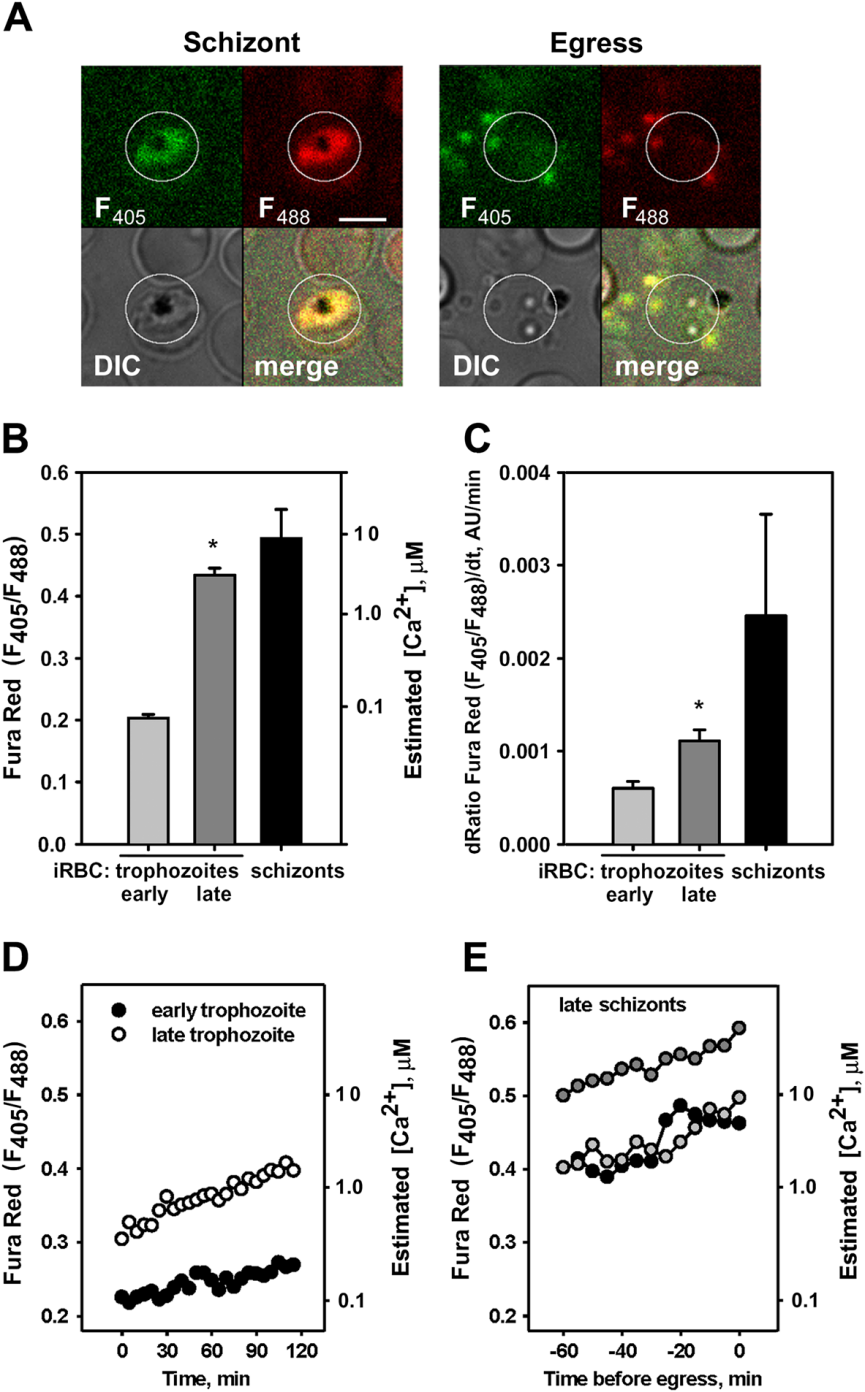
**Free Ca**^**2+ **^**content and free Ca**^**2+ **^**kinetics within infected cells labelled with a ratio-metric calcium probe Fura Red AM.** Cells were labelled with Fura Red AM (5 μM) and monitored at 37°C in medium supplemented with 40 μM probenecid. **A**. Fluorescence and DIC images of labelled schizont, four images on left; parasite egress from the same schizont captured in all three channels, four images on right. Circle indicates an area of interest selected for analysis of fluorescence in the two ratio-metric channels. Bar = 5 μm. **B**. Free calcium content in infected cells for three different stages of parasite maturation: early trophozoites, late trophozoite and pre-egress schizonts. Cells were labelled with ratio-metric probe Fura Red AM (5 μM), and monitored at 37°C in the medium supplemented with 40 μM probebecid. Number of analysed cells: early trophozoites – 80, late trophozoites – 60, schizonts – 10. [Ca^2+^] estimation described in the Methods. **C**. Rate of calcium increase observed in the three different stages of parasite maturation described in **B** using the same labelling protocol. Number of analysed cells: early trophozoites – 25, late trophozoites – 60, schizonts – 10. Data presented as a mean ± SEM; significance evaluated using a paired t-test. A statistically significant differences (*) with P < 0.01 was found between early and late trophozoites. **D**. Representative time courses for the calcium increases observed in early trophozoite (filled circles) and late trophozoite (open circles). **E**. Time courses for the calcium increases observed in three pre-egress schizonts (circles filled with black, light gray or dark gray colour for each cell). Note that the ratio could plateau prior to egress (black circles curve).

Probenecid-containing medium was used for these experiments to prevent dye extrusion from parasites [40]. In some recordings a gradual increase in the Fluo-4 fluorescence signal that precedes the fluorescence transients due to erythrocyte membrane poration was observed (see example in Figure 1B; note that the gradual fluorescence increase coincides with the beginning of the host cell shape transformation). To exclude the effect of volume dependent morphological transformations occurring in infected cells approaching egress on the Fluo-4 fluorescence signal and to directly compare cells, the next set of experiments was designed to image schizonts in the presence of probenecid using the ratio-metric Ca^2+^ probe Fura Red AM. The use of a ratio-metric probe mitigates measurement concerns that arise from volume variability, probe incorporation and hydrolysis of AM ester groups, and photobleaching [36]. In addition, image acquisition was switched from laser-scanning confocal microscopy to wide field, EMCCD camera based, microscopy; exposure time was reduced to 200 msec per image. Infected cells were labelled with Fura Red AM (5 μM) and imaged over a 2–4 h period in medium supplemented with probenecid; for each cell, 24–48 frames were acquired at 5-min intervals. To capture the temporal changes in Ca^2+^ Fura Red fluorescence was measured within the parasite compartment (Figure 2A, ROI represented by the white circle) and calculated the fluorescence ratio F_405_/F_488_ for individual infected cells at different stages of parasite maturation. [Ca^2+^ was lowest in early trophozoites and significantly higher (P < 0.01) in late trophozoite and pre-egress schizonts (Figure 2B); this is consistent with previous results [17,41]. Changes in [Ca^2+^ with time were also significantly higher (P < 0.01) in late trophozoites and pre-egress schizonts compared with early trophozoites (Figure 2C-E). Overall, gradual [Ca^2+^ increases were observed in 92% of early trophozoites and 95% of late trophozoites. All the cells that reached egress had elevated [Ca^2+^ levels prior to poration (in the range between 1 and 10 μM). The [Ca^2+^ time course varied, with seven out of 10 schizonts having a continuously increasing [Ca^2+^, while three cells showed a plateau in [Ca^2+^. These data demonstrate an accelerated [Ca^2+^ increase within infected cells at the end of the cycle that culminates in a rapid entry of extracellular calcium following membrane poration seconds before egress.

### Buffers to intracellular, but not extracellular Ca^2+^ prevent parasite egress from erythrocytes

**Figure 3:**
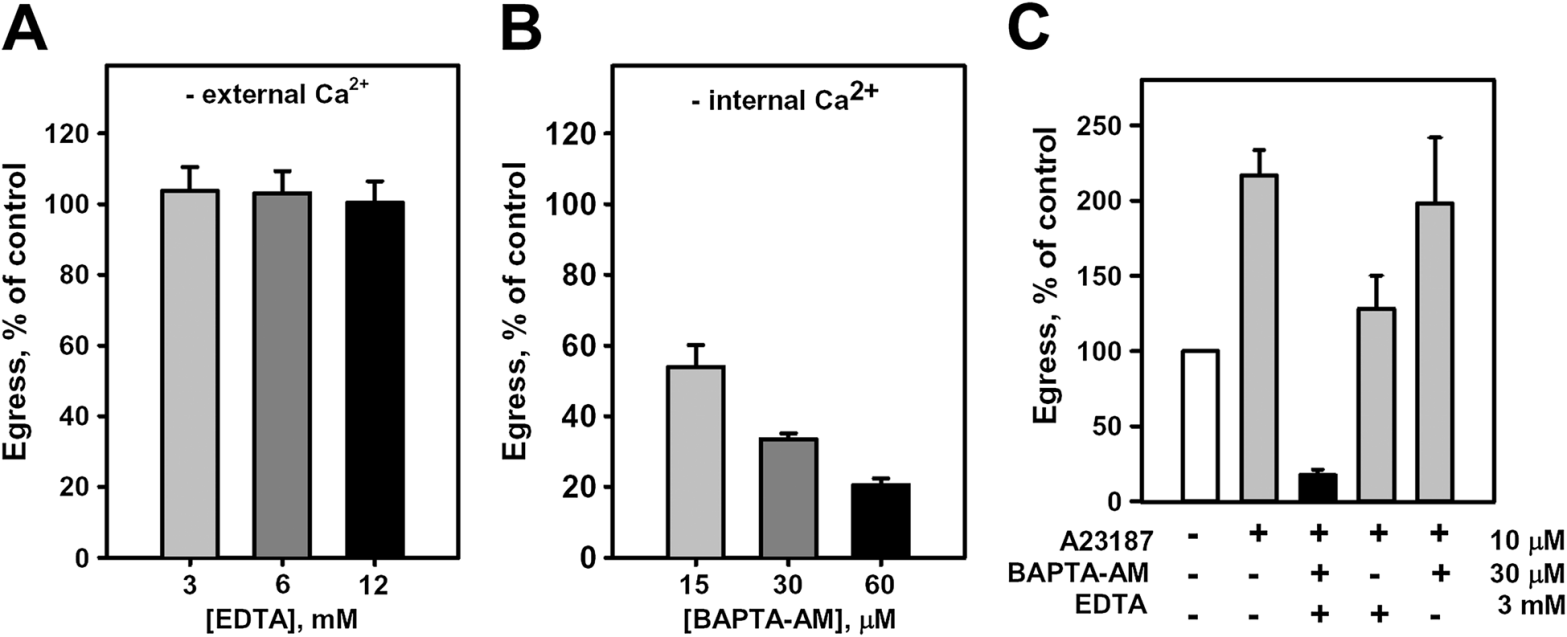
**Manipulating the free calcium concentration in schizonts affects parasite egress. A**. Reducing the extracellular free Ca^2+^ concentration does not affect parasite egress. Synchronized cell cultures approaching the end of the cycle were pretreated with different concentrations of EDTA, pH 7.4, for 5 min at 37°C to chelate external Ca^2+^ and then observed. Drug-pretreated and control cultures were kept for two hours at 37°C to accumulate sites of egress and then egress was evaluated as described in the Methods and presented as Mean ± SEM, n = 3-4 independent experiments; 7,429 infected cells were analyzed. Concentration of free calcium was less than 10 nM according to a fluorimetric assay for free Ca^2+^. **B**. Reduction in intracellular Ca^2+^ concentration by BAPTA inhibits parasite egress. Synchronized cell cultures approaching the end of the cycle were pretreated outside the chambers with increasing concentrations of the cell permeant Ca^2+^ chelator BAPTA AM at 37°C for 30 min. Pretreated cultures were then injected into the chambers for microscopy and kept at 37°C for 90 min to accumulate sites of egress and then parasite egress was assessed in treated and control cultures as described above (mean ± SEM, n = 3-4 independent experiments; 9,669 infected cells were analyzed). **C**. The Ca^2+^ ionophore A23187 activates parasite egress only in the presence of intra-and extra-cellular Ca^2+^ and upon short (15–30 min) treatment of infected cells. Cells pretreated with EDTA (5 min at 37°C) or BAPTA AM (30 min at 37°C) or both were injected into the chambers immediately after addition of ionophore into the medium. Chambers were kept at 37°C for 15–30 min to accumulate sites of egress and then parasite egress was assessed in cultures as described above (mean ± SEM, n = 2-6 independent experiments, at least 300 infected cells were analyzed for each condition in each experiment).

Next, the compartment contributing to the slow rise in intracellular [Ca^2+^, and the effect on egress of reagents interfering with calcium fluxes was determined. The existing indirect observations that external Ca^2+^ does not affect parasite egress [21] were quantitatively confirmed and the time frame for this independence on extracellular calcium was established. The culture medium bathing synchronized schizonts was depleted of free calcium (0.4 mM) for 2 hr using an excess (up to 12 mM) of the Ca^2+^ and Mg^2+^ chelator, EDTA. There was no significant effect on the parasite cycle evaluated using both the percentage of egress (Figure 3A) and the observed morphological transformations of the infected erythrocytes preceding egress. The same result was obtained in two independent experiments with 3 mM EGTA, a chelating agent with higher affinity for Ca^2+^ than Mg^2+^; mean egress was equal to the control value. In addition, parasite egress comparable to control (Additional file 2, A) was observed in a Ca^2+^-free, isotonic salt solution supplemented with glucose and AlbuMax II, two obligate medium components for viable schizonts. Thus, the last two hours of the erythrocyte cycle of *P. falciparum,* a time frame that spans the end of parasite morphogenesis and parasite egress [42], are independent of external free calcium. These data also imply that entry of Ca^2+^ through the erythrocyte plasma membrane is not required for parasite egress. This is particularly interesting in light of the total dependence of the poration signal on extracellular Ca^2+^ determined above. Thus, the entry of extracellular Ca^2+^ following erythrocyte membrane poration preceding host cell membrane rupture is not essential in finalizing the parasite egress programme.

The independence on external Ca^2+^ during the last two hours of the parasite cycle, coupled with the finding of increases in intracellular [Ca^2+^ during that same time period suggests two possibilities: that Ca^2+^ is not required for the observable parasite egress stages, i e, swelling of parasitophorous vacuole, poration of host cell membrane and digestion of erythrocyte cytoskeleton, or that intracellular Ca^2+^ regulates the entire egress pathway. The role of internal Ca^2+^ stores in parasite egress was tested by treating cells with the membrane permeant Ca^2+^ chelator BAPTA AM to minimize intracellular increases in [Ca^2+^. Unlike external Ca^2+^ chelation, BAPTA AM inhibited egress in a dose-dependent manner over a micro-molar concentration range (15–60 μM) in the bathing media (Figure 3B). To exclude a known nonspecific effects of BAPTA AM on cell physiology, ATP depletion following the production of formaldehyde upon hydrolysis of AM esters [43] and the finding that this effect is minimized by the addition of exogenous pyruvate [44], parasite egress was assessed in the presence of both BAPTA AM (30 μM) and pyruvate (4–12 mM). BAPTA AM again inhibited parasite egress in the presence of pyruvate (Additional file 2, B). Moreover, calcein AM, a vital dye that labels both parasite and erythrocyte cytosol following AM ester hydrolysis, did not affect parasite egress when added to the culture medium in the same concentrations as BAPTA AM (Additional file 2, C). The results of these control experiments indicate that the effect of BAPTA AM on parasite egress is due to the Ca^2+^ affinity of BAPTA, and support the hypothesis that increases in intracellular [Ca^2+^ are required for parasite egress. To further test this hypothesis, using the Ca^2+^ ionophore A23187, a permeability pathway for exogenous Ca^2+^ to enter schizonts saturated with BAPTA was created. This treatment rescued BAPTA-blockage of parasite egress at all times during the last 45–60 min of the parasite cycle (Figure 3C the first bar on the right, Additional file 2, D) confirming that intracellular free Ca^2+^ is needed for egress.

If only one stage of egress was inhibited, one would expect a pile-up of schizonts at that one stage of egress, and no effect of Ca^2+^ chelation on schizonts whose egress pathway had progressed beyond the putative Ca^2+^-sensitive stage. To test this reasoning, live-cell microscopy was used to examine the morphology of infected cells treated with BAPTA AM. Schizonts whose cycle was blocked by Ca^2+^ chelation were morphologically indistinguishable from untreated schizonts, suggesting that their morphogenesis was not affected by BAPTA AM. They did not enter into the stage of shape transformations that precedes egress and produces a “flower” form of schizonts, i e, swelling of the parasitophorous vacuole, shrinkage of the erythrocyte cytoplasm, and erythrocyte membrane poration [6,7,45]. The duration of pre-egress shape-transformations in control schizonts lasted less than 10 min (6.8 ± 0.6 min; mean ± SEM, n = 55). However, in eight of 10 mature schizonts treated with 60 μM BAPTA AM and randomly selected for light microscopy observation the expected cycle progression towards parasite egress was not detected over relatively long observation times (up to 37 min of observation; Additional file 3). This data suggests that Ca^2+^ chelation blocks the egress pathway upstream of vacuole swelling. Based on the shortest effective inhibitory time of BAPTA treatment (45 min, Additional file 2, D), and prevention of the flower stage in BAPTA-treated schizonts (the last 10 min of the cycle), it is reasonable to conclude that the earliest Ca^2+^-dependent stages in the egress programme occurs between the last 45 and 10 minutes of the parasite cycle.

### The source of Ca^2+^ for egress

Next, the hypothesis that membrane-bound compartments within the schizont were the source of intracellular Ca^2+^ needed to increase cytoplasmic [Ca^2+^ to the threshold levels required for egress initiation and progression was tested. The prediction was that increasing the Ca^2+^ permeability of internal membranes in the absence of extracellular Ca^2+^ would prematurely increase cytoplasmic [Ca^2+^ and prematurely initiate egress. Synchronized cultures of schizonts were exposed to the Ca^2+^ ionophore A23187 in the presence or absence of extracellular Ca^2+^. Indeed, in both cases significantly accelerated egress of parasites was detected (Figure 3C). Morphological analysis of cultures treated with ionophore did not reveal any differences in the sites of parasite egress, i e, limited areas in microscopy chambers where scattered parasites, food vacuole, fragmented host membrane are localized [6,34]. There was no significant difference in the viability of parasites released in the presence of A23187 compared to control (2.07 ± 0.48% of dead cells *vs* 0.85 ± 0.28% respectively, ethidium homodimer assay, mean ± SEM, n = 369 and 367 respectively; P = 0.1). The accelerated egress was dose-dependent in the 1–10 μM ionophore concentration range (Additional file 4, A). Note, the active concentration of A23187 may be even lower because this hydrophobic ionophore is known to bind to serum albumin in the medium [46]. The finding that accelerated egress occurs in the absence of extracellular Ca^2+^ (Figure 3C) indicates that an intracellular Ca^2+^ source is sufficient for initiating egress. To confirm that egress initiation by A23187 is Ca^2+^-dependent, cells were treated with ionophore in Ca^2+^-free medium (medium with 3 mM EDTA) following chelation of intracellular Ca^2+^ stores with 30 μΜ BAPTA AM. Parasite egress was inhibited (Figure 3C), indicating the finite nature of the chelatable Ca^2+^ pool within the internal stores since A23187 plus exogenous Ca^2+^ could rescue BAPTA AM treated cells (Figure 3C). Thus, finite internal stores of Ca^2+^ can initiate and regulate the egress pathway.

**Figure 4:**
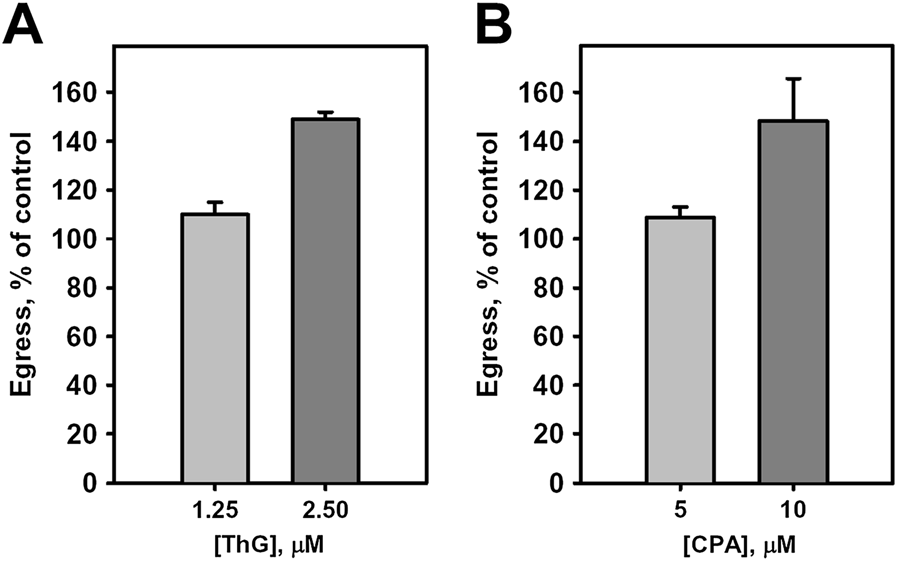
**Acceleration of parasite egress induced by inhibitors of the *Plasmodium *Ca**^**2+ **^**pump PfATP6.** Cells were treated for 30 min at 37°C in chambers. **A**. ThG – Thapsigargin, mean of two independent experiments, total number of analysed infected cells, 3,300. **B**. CPA – cyclopiazonic acid, mean ± SEM, n = 3 independent experiments, total number of analysed cells, 3,600.

A likely compartment for the intracellular Ca^2+^ store activating egress is the endoplasmic reticulum of the *de novo* produced parasites, which is controlled by the malarial SERCA orthologue PfATP6 [47,48]. If true, then treatment of mature schizonts with inhibitors of PfATP6 should elevate [Ca^2+^ in the parasite cytoplasm, mimic constitutive egress initiation, and result in the acceleration of parasite egress. Indeed, egress acceleration was observed following 30-min treatment of schizonts with thapsigargin (ThG) and cyclopiazonic acid (CPA), two known inhibitors of PfATP6 [47,48] (Figure 4A-B). These results are consistent with the assignment of the schizont ER as an intracellular Ca^2+^ store for the egress pathway. The results described above using ThG and CPA are the first demonstration that malaria parasite egress can be *induced* pharmacologically, compared to previous reports where malaria parasite egress was diminished using protease inhibitors (reviewed in [49]). The enhancement of this stage of the malaria asexual replication cycle raises the worrisome possibility that the clinical course of a patient with malaria may be worsened due to side effects of therapeutics administered for various reasons.

One of the suggested targets for intracellular Ca^2+^ in the egress programme is the Ca^2+^-dependent protein kinase PfCDPK5 [5] that is activated downstream of another kinase involved in egress, a cyclic guanosine monophosphate (cGMP)–dependent kinase PfPKG [8]. If PfCDPK5 were a required transducer of the rise in [Ca^2+^ to affect release, then one would predict that inhibiting kinase activity during the sensitivity time to increased [Ca^2+^ would prevent egress. To test this prediction, schizonts were treated with 1.0-2.5 μM staurosporine, a promiscuous inhibitor of protein kinases [50] during the last hour of the parasite cycle. Staurosporine strongly inhibited parasite egress (82.8 ± 1.7% inhibition; mean ± SEM, n = 4). This pharmacological data suggests that the Ca^2+^-sensitive stage of parasite egress and the stage of egress that requires kinase activities are temporally overlapping events.

### Initiation of vacuole swelling is a newly discovered Ca^2+^ target in egress execution

**Figure 5:**
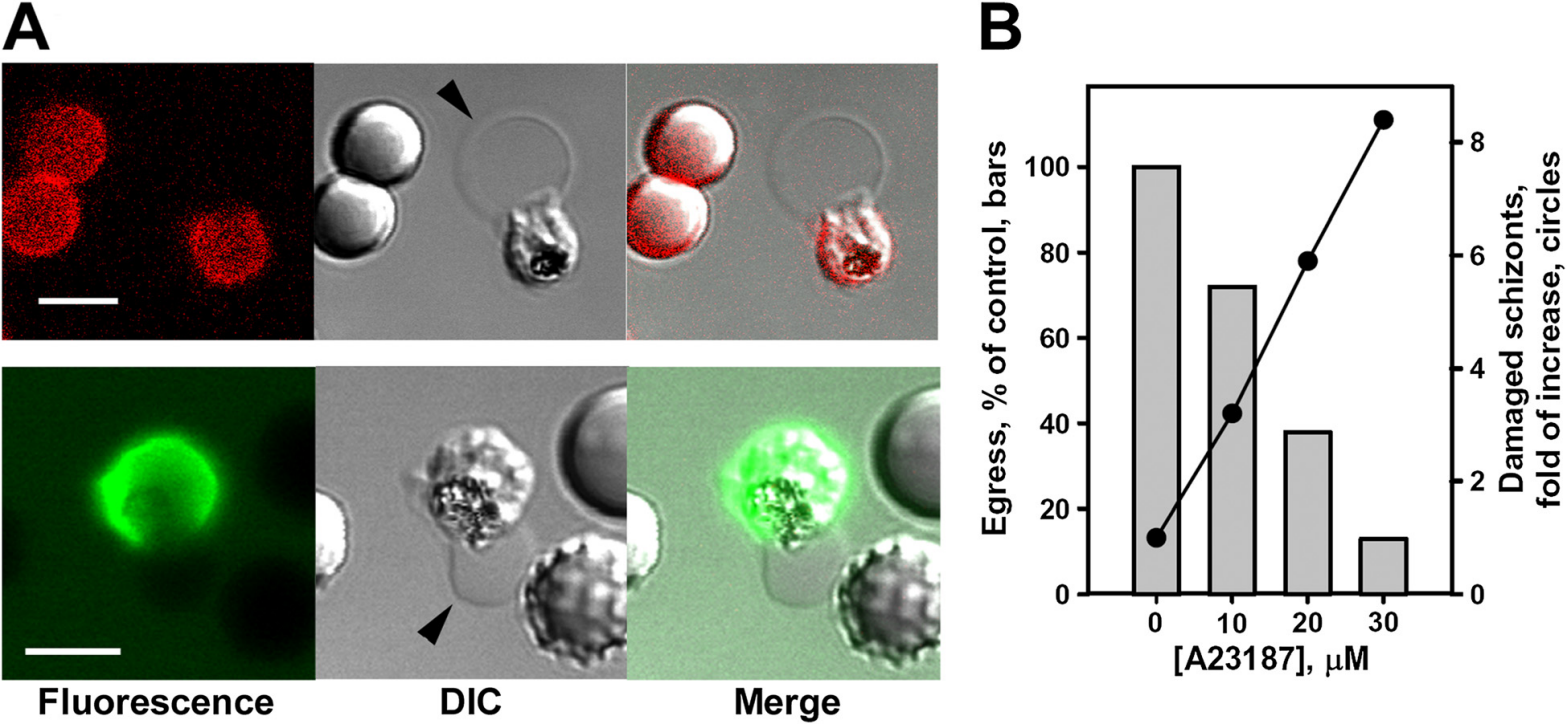
**A23187-induced and Ca**^**2+ **^**-dependent parasite death. A**. Immature schizonts from infected erythrocytes critically swelled with extruded parasitophorous vacuoles. Cultures were treated with 10 μM A23187 in chambers for two hours in the presence of fluorescent antibodies to the major erythrocyte surface protein glycophorin A (red colour in upper images) and with fluorescent phalloidin (green colour in lower images) to detect the major erythrocyte cytoskeletal protein F-actin. Note the unlabelled membranes of the parasitophorous vacuoles (black arrowheads) extruded from the labelled erythrocyte membranes; bar = 5 μm. **B**. Extended treatment (2 hours) of cultures with increased concentrations of ionophore inhibited parasite egress and led to accumulation of damaged schizonts (a representative experiment, mean of three measurements, total number of infected cells analysed, 1,402).

Light microscopy of pre-egress schizonts revealed dynamic changes in infected erythrocyte morphology that lead to the formation of a “flower” form of schizont wherein mature parasites are situated in the swelled parasitophorous vacuole, while the host cell compartment is visibly reduced [6,7,45]. Pre-egress parasitophorous vacuole swelling and the sensitivity of egress to the osmotic pressure of the medium [6] suggest that ion and water redistribution in infected cells controls egress. Since depletion of intracellular Ca^2+^ blocks the cycle prior to PV swelling, a hypothesis that the detected increase in intracellular [Ca^2+^ is sufficient for triggering PV swelling was tested. Using light microscopy, the morphology of infected cells treated with the calcium ionophore A23187 was analysed. As expected, normal erythrocytes were crenated due to activation of the Gardos channel, [51] leading to cell dehydration (Additional file 4, B; lower left cell). The most profound morphological abnormalities were observed in the population of A23187-treated immature schizonts. They exhibited a swollen parasitophorus vacuole that was frequently extruded from haemolysed erythrocytes (Figure 5A). The swelling of the vacuole blocked parasite development and, as a result, a dose-dependent inhibition of parasite egress in cultures treated with A23187 for prolonged periods of time accompanied by accumulation of damaged schizonts was observed (Figure 5B). In contradistinction, in mature schizonts the A23187-accelerated PV swelling mimics the normal swelling of the vacuole during egress execution [7] and suggests yet another target for increases in intracellular [Ca^2+^: the activation of ionic permeability changes in the vacuolar membrane that leads to visible reduction of the erythrocyte compartment and swelling of the parasitophorous vacuole.

*Plasmodium* thus is similar to other *Apicomplexan* parasites with respect to the involvement of Ca^2+^ in its egress. A23187 is a potent trigger of *Toxoplasma* parasite egress, where the effect is also independent of external Ca^2+^[24], but it is dependent on internal Ca^2+^ that activates two kinases involved in egress, TgCDPK1 and TgCDPK3 [29] . In addition, A23187 inducible egress is also seen in two other *Apicomplexan* parasites of veterinary significance: *Neospora caninum* and *Eimeria bovis*[52]. A distinct difference between *Toxoplasma gondii*, *N. caninum, E. bovis* and results presented in this study with *P. falciparum* is the timing of the ionophore-induced egress: in the first three organisms egress could be induced as early as two hours after parasite invasion [52,53], while in malaria parasite egress required maturation – full completion of merozoites morphogenesis within the schizonts – to prevent premature death in the host cell. This difference in parasite biology allowed the establishment of an underlying mechanism for A23187-induced parasite egress, which is the swelling of the parasitophorous vacuole. There are other indications that *Plasmodium* and *Toxoplasma* egress pathways differ: *Toxoplasma* species initiate egress by production of the plant hormone abscisic acid (ABA) [28] and the progression of unaffected egress in *Toxoplasma* depends on Ca^2+^-binding protein DOC2 [30]. However, the ABA pathway has not been confirmed for *P. falciparum* (S. Glushakova, unpublished observation) and the DOC2 protein is not involved in the egress pathway of *Plasmodium*[30]. These differences in parasite biology highlight the divergence between *Plasmodium* and *Toxoplasma* and stress the importance of cautious extrapolation of data on the egress mechanism of *Toxoplasma* to *Plasmodium* and *vice versa*.

### Ca^2+^-dependent digestion of host-cell cytoskeleton

The ionophore-induced acceleration of malaria parasite egress could not be accomplished without dismantling the host erythrocyte cytoskeleton [4,54]. Cytoskeleton digestion precedes constitutive egress [54] and could be blocked by inhibitors of cysteine proteases [45,54]. Presented here findings that the sites of parasite egress in the ionophore-treated cultures are indistinguishable from those in untreated egress and the presence of fragmented erythrocyte membranes supports the hypothesis that the erythrocyte cytoskeleton is dismantled by Ca^2+^-activation of calpain [4]. In addition, the observation of blebbed erythrocyte membranes shed from damaged, ionophore treated, immature schizonts suggests that Ca^2+^ may have activated calpain in these cells as well (Additional file 4, C).

### Unresolved questions

Further investigations are required to establish the source of free Ca^2+^ for initiation of vacuole swelling and erythrocyte cytoskeleton digestion and the pathway by which this second messenger arrives at the site of action. At the onset of egress, both host and parasite cells, each have their individual cytoplasm low in [Ca^2+^. Since putative targets for [Ca^2+^ are in both cell’s cytoplasm, it is reasonable to think that each cytoplasm can receive calcium. The parasite compartment includes two potential stores, parasite’s internal stores and the parasitophorous vacuole with a relatively high [Ca^2+^[13]. The host erythrocyte cytoplasm borders the parasitophorous vacuole, with its irregular extensions into the erythrocyte cytoplasm [55]. In this speculation the parasitophorous vacuole is associated with the calcium storage function usually seen in the endoplasmic reticulum. Another unknown is the nature of the effector proteins involved in vacuolar membrane swelling. Usually, it is a change in the permeability of a limiting membrane that drives biological swelling via the resulting osmotic imbalance, implicating ion channels and transporters of either parasite or host cell origin. If involved, these proteins are attractive therapeutic target molecules and may be the basis for the design of new anti-malarial drugs.

## Conclusion

In this study an increase in cytoplasmic [Ca^2+^] that appears to be essential for progression of the emerging release process of *P. falciparum* was detected. Intracellular [Ca^2+^] has multiple targets in the malaria parasite egress programme: initiation of egress (signalling) within the last hour of the cycle through Ca^2+^ release from the ER stores of mature schizonts and following activation of Ca^2+^-dependent PfCDPK5, induction of Ca^2+^-dependent vacuole swelling within the last 10–20 min of cycle, and activation of Ca^2+^-dependent host-cell calpain for destabilization of the erythrocyte cytoskeleton finalized prior to egress (Additional file 5). The finding that malaria parasite egress from erythrocytes could be pharmacologically induced by modulators of cellular Ca^2+^ homeostasis was demonstrated for the first time and thus these reagents now could be used to study egress pathway in a controlled manner.

A modified egress pathway is now presented. Parasites replicate within a single plasma membrane. This membrane invaginates wrapping newly synthesized material and creating distinct merozoites. Upon completion of morphogenesis, Ca^2+^ signalling from intracellular stores initiates a cascade of biochemical and physiological events that initiate parasite departure from the host cell. This Ca^2+^-dependent cascade includes the activation of a protease cascade (reviewed in [49]) and at least one Ca^2+^-dependent protein kinase [5]. For the execution of egress, increases in [Ca^2+^ activates vacuole swelling, presumably via Ca^2+^- activated channels to alter internal membrane permeability needed to rupture the parasitophorus vacuole. The same intracellular Ca^2+^ must activate calpain and the suggested perforin in the erythrocyte cytoplasmic compartment to destabilize and perforate the host cell membrane [4,7,45,54]. Cytoskeleton digestion eliminates erythrocyte membrane resistance to host cell shrinkage and permeation of erythrocyte membrane reduces the haemoglobin concentration and minimizes erythrocyte cytoplasm viscosity. Egress of parasites from the bursting vacuole through the haemoglobin-depleted erythrocyte compartment and weakened erythrocyte membrane into the bloodstream finalizes the erythrocyte cycle of this human pathogen.

## Abbreviations

ATP: Adenosine triphosphate; BAPTA: Glycine N,N’-[1,2-ethanediylbis(oxy-2,1-phenylene)]bis[N-[2-[(acetyloxy)methoxy]-2-oxoethyl]] bis[(acetyloxy)methyl] ester; cGMP: Cyclic guanosine monophosphate; CPA: Cyclopiazonic acid; EDTA: Ethylene diamine tetraacidic acid; EGTA: Ethylene glycol tetraacidic acid; EMCD: Electron multiplying charge coupled device; ER: Endoplasmic reticulum; Kd: Dissociation constant; LSM: Laser scanning microscope; NA: Numeric aperture; NIH: National Institutes of Health; PfATP6: *Plasmodium falciparum* Ca^2+^-ATP-ase 6; PfCDPK5: *Plasmodium falciparum* calcium-dependent protein kinase 5; PfPKG: *Plasmodium falciparum* cGMP-dependent protein kinase; PV: Parasitophorous vacuole; ThG: Thapsigargin; TIRF: Total internal reflection fluorescence microscopy; SEM: Standard error of the mean; SERCA: Sarco/endoplasmic reticulum Ca^2+^-ATP-ase.

## Competing interests

The authors declare that they have no competing interests.

## Authors’ contributions

JZ, SG and VL conceived and designed the experiments. SG and VL performed the experiments. SG, VL, PSB, KM, GH and JZ analysed the data. JZ, SG, PSB, VL, GH and KM wrote the paper. All authors read and approved the final manuscript.

## Supplementary Material

Additional file 1 **Free calcium kinetics in schizonts undergoing parasite egress.** The data provided show the variability in free calcium kinetics observed in schizonts undergoing parasite egress. Cells were labeled with Fluo-4 AM (5 μM), and monitored at 37°C in full medium. A sharp increase in fluorescence reflects an influx of free calcium from the medium into the cell through pores formed in the erythrocyte membrane. Egress occurred in the frame marked by an arrow. Different kinetic behaviors are observed: stable **(A, B)** or steadily increasing **(C, D)** fluorescence prior to membrane poration and parasite egress; sharp **(A, C, D)** or steady **(B)** drops in fluorescence to the background level reflecting leakage of Fluo-4 from erythrocytes following membrane poration but before membrane rupture. Click here for file

Additional file 2 **Chelation of internal but not external Ca**^**2+**^**inhibits parasite egress.** The data provided show control experiments that tested the effect of external and internal free calcium chelation on parasite egress. **A.** Parasite egress proceeds normally in a Ca^2+^ free isotonic salt solution (PBS) supplemented with glucose and AlbuMax II. In some experiments EDTA (3 mM) was added to reduce trace amounts of free calcium in the AlbuMax II solution. Cultures were treated 30–60 min at 37°C in chambers. Control cultures were maintained in complete medium (mean ± SEM, n = 3-5). **B.** Inhibition of parasite egress by BAPTA AM does not depend on ATP-depletion in erythrocytes. Cells were pretreated 30 min at 37°C in media with 30 μM BAPTA AM and different concentrations of Na-pyruvate and then incubated in chambers for 90 min at 37°C. Control cultures were incubated in medium without BAPTA AM and Na-pyruvate. An individual experiment, mean of four measurements. **C.** Hydrolysis of the AM ester in cells labeled with calcein AM does not affect parasite egress. Cultures were pretreated 30 min at 37°C in the presence of calcein AM or BAPTA AM and then incubated 30 additional minutes at 37°C in the chamber (mean ± SEM, n = 3). Bars: BAPTA AM, black; calcein AM, grey. DIC (upper image), calcein fluorescence (green) and merged images of calcein-labeled infected and normal erythrocytes. Bar = 5 μm. **D.** Chelation of intracellular calcium by BAPTA within the last 45–60 min of the parasite cycle inhibits parasite egress. Cultures were pretreated 30 min at 37°C in the presence of 30 μm BAPTA AM and then incubated 15 or 30 additional minutes in the chamber (45 min treatment, mean of two independent experiments; 60 min treatment, mean ± SEM, n = 7). Click here for file

Additional file 3 **Depletion of intracellular calcium blocks cycle progression upstream of the morphological transformations of infected erythrocytes that precede parasite egress.** The data provided show light microscopy images of BAPTA AM treated cells. BAPTA AM treatment blocks progression of schizont into the schizont “flower” form characterized by a swelled parasitophorous vacuole and reduced erythrocyte cytoplasm volume. Mature schizonts were pretreated with 60 μM BAPTA AM (30 min at 37°C) and analysed using light microscopy. Randomly selected schizonts do not demonstrate the expected cycle progression towards parasite egress over relatively long observation times (up to 37 minutes of observation). Bar = 5 μm. Click here for file

Additional file 4 **Effect of calcium ionophore A23187 on parasite egress, cell morphology and erythrocyte membrane of infected cells.** The data provided show additional experimental results on the effect of A23187 on parasite egress and morphology of treated cells. **A.** Activation of parasite egress upon short-time treatment of schizonts with calcium ionophore A23187 is dose-dependent. Culture medium was supplemented with different concentrations of A23187 and cells were then placed in the chamber for 30 min incubation at 37°C. Parasite egress in treated and control cultures was assessed as described in the Methods (combined data from two independent experiments; mean ± SEM of three measurements). **B.** Marked differential morphological changes in ionophore-treated cells. Normal erythrocytes were crenated (lower left cell), mature schizont was accelerated to egress and has blebbed erythrocyte membrane (upper left cell) and trophozoite appeared ballooned due to the swelling of the parasitophorous vacuole (cell on the right). Bar = 5 μm. **C.** Ionophore-induced shading and blebbing of erythrocyte membrane in immature schizont. Blebbed erythrocyte membrane (white arrowhead) shaded from the immature schizont (black arrowhead) damaged by ionophore treatment suggesting that Ca^2+^ fluxes activated calpain and cytoskeleton digestion in this cell. Green colour - erythrocyte actin cytoskeleton labeled with fluorescent phalloidin-Alexa 488. Bar = 5 μm. Click here for file

Additional file 5 **Suggested Ca**^**2+**^**-dependent steps in parasite egress programme.** A table-style presentation of experimental data on the involvement of Ca^2+^ in the parasite egress programme. Click here for file
