# Supplementary figures and images for: Cytoplasmic free Ca2+ is essential for multiple steps in malaria parasite egress from infected erythrocytes
Source: Malar J. 2013 Jan 30;12:41. doi: 10.1186/1475-2875-12-41 (PMC3564835; doi:10.1186/1475-2875-12-41)

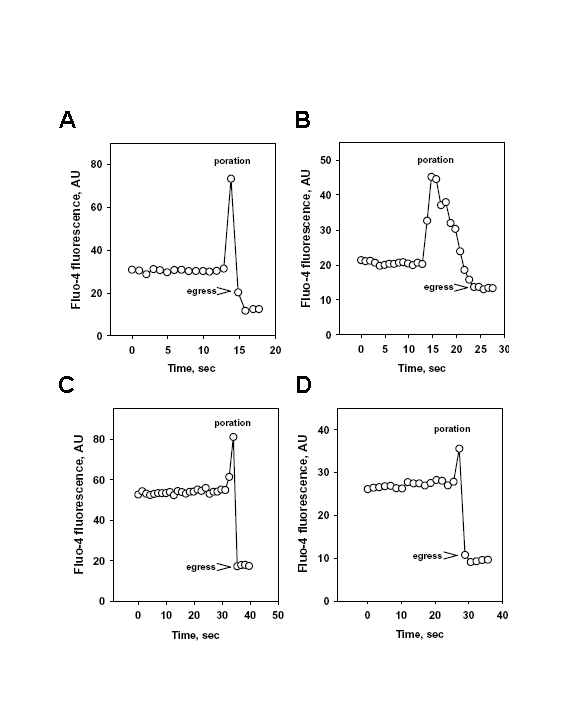

Supplement: Additional file 1 — Free calcium kinetics in schizonts undergoing parasite egress. The data provided show the variability in free calcium kinetics observed in schizonts undergoing parasite egress. Cells were labeled with Fluo-4 AM (5 μM), and monitored at 37°C in full medium. A sharp increase in fluorescence reflects an influx of free calcium from the medium into the cell through pores formed in the erythrocyte membrane. Egress occurred in the frame marked by an arrow. Different kinetic behaviors are observed: stable (A, B) or steadily increasing (C, D) fluorescence prior to membrane poration and parasite egress; sharp (A, C, D) or steady (B) drops in fluorescence to the background level reflecting leakage of Fluo-4 from erythrocytes following membrane poration but before membrane rupture. [file 1475-2875-12-41-S1.tiff]

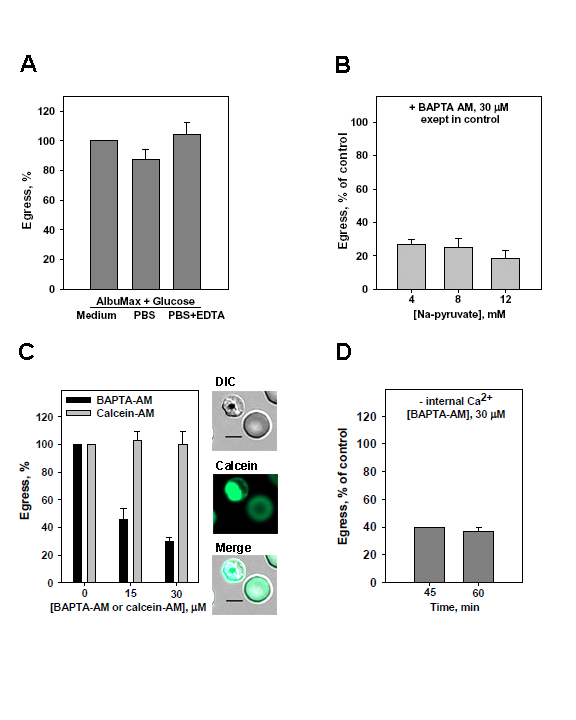

Supplement: Additional file 2 — Chelation of internal but not external Ca2+inhibits parasite egress. The data provided show control experiments that tested the effect of external and internal free calcium chelation on parasite egress. A. Parasite egress proceeds normally in a Ca2+ free isotonic salt solution (PBS) supplemented with glucose and AlbuMax II. In some experiments EDTA (3 mM) was added to reduce trace amounts of free calcium in the AlbuMax II solution. Cultures were treated 30–60 min at 37°C in chambers. Control cultures were maintained in complete medium (mean ± SEM, n = 3-5). B. Inhibition of parasite egress by BAPTA AM does not depend on ATP-depletion in erythrocytes. Cells were pretreated 30 min at 37°C in media with 30 μM BAPTA AM and different concentrations of Na-pyruvate and then incubated in chambers for 90 min at 37°C. Control cultures were incubated in medium without BAPTA AM and Na-pyruvate. An individual experiment, mean of four measurements. C. Hydrolysis of the AM ester in cells labeled with calcein AM does not affect parasite egress. Cultures were pretreated 30 min at 37°C in the presence of calcein AM or BAPTA AM and then incubated 30 additional minutes at 37°C in the chamber (mean ± SEM, n = 3). Bars: BAPTA AM, black; calcein AM, grey. DIC (upper image), calcein fluorescence (green) and merged images of calcein-labeled infected and normal erythrocytes. Bar = 5 μm. D. Chelation of intracellular calcium by BAPTA within the last 45–60 min of the parasite cycle inhibits parasite egress. Cultures were pretreated 30 min at 37°C in the presence of 30 μm BAPTA AM and then incubated 15 or 30 additional minutes in the chamber (45 min treatment, mean of two independent experiments; 60 min treatment, mean ± SEM, n = 7). [file 1475-2875-12-41-S2.tiff]

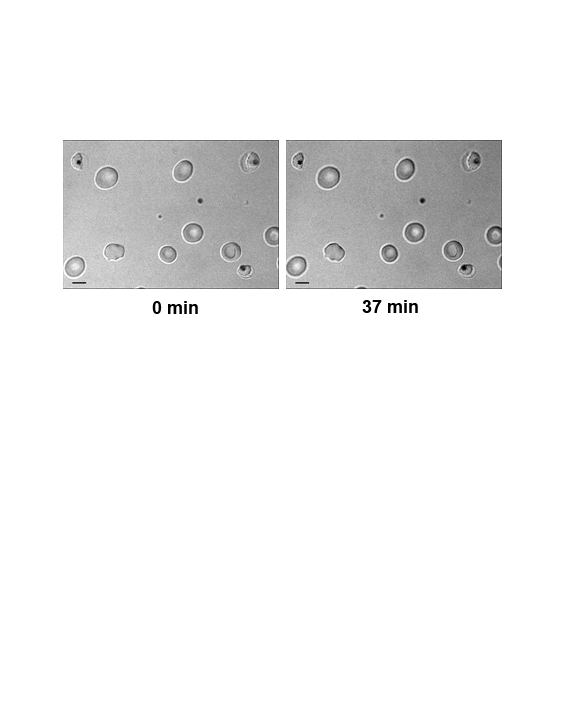

Supplement: Additional file 3 — Depletion of intracellular calcium blocks cycle progression upstream of the morphological transformations of infected erythrocytes that precede parasite egress. The data provided show light microscopy images of BAPTA AM treated cells. BAPTA AM treatment blocks progression of schizont into the schizont “flower” form characterized by a swelled parasitophorous vacuole and reduced erythrocyte cytoplasm volume. Mature schizonts were pretreated with 60 μM BAPTA AM (30 min at 37°C) and analysed using light microscopy. Randomly selected schizonts do not demonstrate the expected cycle progression towards parasite egress over relatively long observation times (up to 37 minutes of observation). Bar = 5 μm. [file 1475-2875-12-41-S3.tiff]

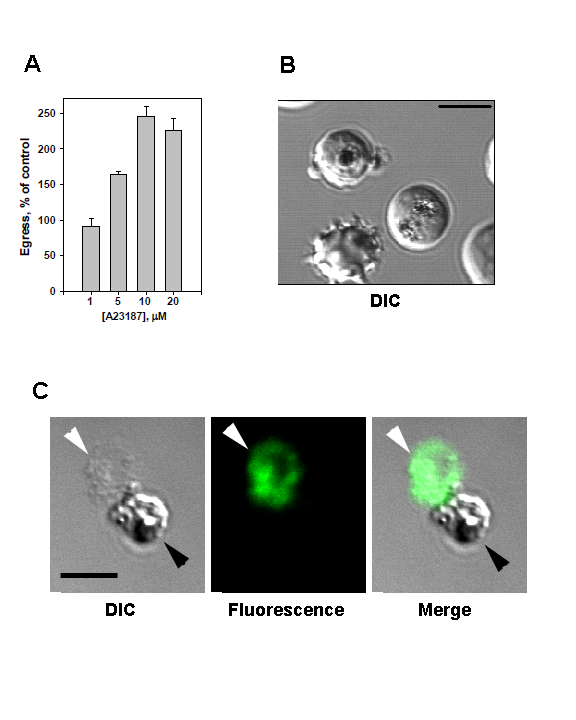

Supplement: Additional file 4 — Effect of calcium ionophore A23187 on parasite egress, cell morphology and erythrocyte membrane of infected cells. The data provided show additional experimental results on the effect of A23187 on parasite egress and morphology of treated cells. A. Activation of parasite egress upon short-time treatment of schizonts with calcium ionophore A23187 is dose-dependent. Culture medium was supplemented with different concentrations of A23187 and cells were then placed in the chamber for 30 min incubation at 37°C. Parasite egress in treated and control cultures was assessed as described in the Methods (combined data from two independent experiments; mean ± SEM of three measurements). B. Marked differential morphological changes in ionophore-treated cells. Normal erythrocytes were crenated (lower left cell), mature schizont was accelerated to egress and has blebbed erythrocyte membrane (upper left cell) and trophozoite appeared ballooned due to the swelling of the parasitophorous vacuole (cell on the right). Bar = 5 μm. C. Ionophore-induced shading and blebbing of erythrocyte membrane in immature schizont. Blebbed erythrocyte membrane (white arrowhead) shaded from the immature schizont (black arrowhead) damaged by ionophore treatment suggesting that Ca2+ fluxes activated calpain and cytoskeleton digestion in this cell. Green colour - erythrocyte actin cytoskeleton labeled with fluorescent phalloidin-Alexa 488. Bar = 5 μm. [file 1475-2875-12-41-S4.tiff]
