# Supplementary material for: Cytoplasmic free Ca2+ is essential for multiple steps in malaria parasite egress from infected erythrocytes
Source: Malar J. 2013 Jan 30;12:41. doi: 10.1186/1475-2875-12-41 (PMC3564835; doi:10.1186/1475-2875-12-41)
Supplement: Additional file 5 — Suggested Ca2+-dependent steps in parasite egress programme. A table-style presentation of experimental data on the involvement of Ca2+ in the parasite egress programme. [file 1475-2875-12-41-S5.pdf]

Additional file 5:

Suggested Ca^{2+}-dependent steps in parasite egress program

Location	Process	Physiological Outcome
**Intraparasitic Ca^{2+}stores**	**Release of free Ca^{2+} from the ER, activation of enzymes involved in parasite egress: PfCDPK5, proteases?**	**Initiation of parasite egress.**
**Parasitophorous vacuole/erythrocyte cytoplasm**	**Ca^{2+} -dependent increase in the PVM permeability to ions: Na^{+} and K^{+}?**	**Vacuole swelling due to ion/water fluxes into the vacuole from the host cytoplasm.**
**Erythrocyte cytoplasm**	**Ca^{2+}-dependent activation of host protease calpain and release of membrane pore-forming agents.**	**Digestion of erythrocyte cytoskeleton, poration of erythrocyte membrane and weakening of erythrocyte membrane stability.**
